# Pathogeneses and Imaging Features of Cerebral White Matter Lesions of Vascular Origins

**DOI:** 10.14336/AD.2021.0414

**Published:** 2021-12-01

**Authors:** Xiaoqin Wu, Jingyuan Ya, Da Zhou, Yuchuan Ding, Xunming Ji, Ran Meng

**Affiliations:** ^1^Department of Neurology, Xuanwu Hospital, Capital Medical University, Beijing, China.; ^2^Advanced Center of Stroke, Beijing Institute for Brain Disorders, Beijing, China.; ^3^Department of China-America Institute of Neuroscience, Xuanwu Hospital, Capital Medical University, Beijing, China.; ^4^Division of Clinical Neuroscience, Queen's Medical Center School of Medicine, the University of Nottingham, Nottingham NG7 2UH, UK.; ^5^Department of Neurosurgery, Wayne State University School of Medicine, Detroit, Michigan 48201, USA

**Keywords:** cerebral white matter lesion, neuroimaging, pathomechanism, cerebral vascular disease

## Abstract

White matter lesion (WML), also known as white matter hyperintensities or leukoaraiosis, was first termed in 1986 to describe the hyperintense signals on T2-weighted imaging (T2WI) and fluid-attenuated inversion recovery (FLAIR) maps. Over the past decades, a growing body of pathophysiological findings regarding WMLs have been discovered and discussed. Currently, the generally accepted WML pathogeneses mainly include hypoxia-ischemia, endothelial dysfunction, blood-brain barrier disruption, and infiltration of inflammatory mediators or cytokines. However, none of them can explain the whole dynamics of WML formation. Herein, we primarily focus on the pathogeneses and neuroimaging features of vascular WMLs. To achieve this goal, we searched papers with any type published in PubMed from 1950 to 2020 and cross-referenced the keywords including “leukoencephalopathy”, “leukoaraiosis”, “white matter hyperintensity”, “white matter lesion”, “pathogenesis”, “pathology”, “pathophysiology”, and “neuroimaging”. Moreover, references of the selected articles were browsed and searched for additional pertinent articles. We believe this work will supply the robust references for clinicians to further understand the different WML patterns of varying vascular etiologies and thus make customized treatment.

The term of white matter lesion (WML), also known as “leukoaraiosis (LA)” or white matter hyperintensity (WMH), was originally reported by Hachinski et al. in 1986 to define the hyperintense signals of cerebral white matter (WM) on T2-weighted imaging (T2WI) and fluid-attenuated inversion recovery (FLAIR) maps [1]. Typically, the patterns of WML may present as multifocal or diffuse lesions with different sizes, shapes and locations. Through decades of unremitting efforts, numerous theories and models have been developed to unravel the pathophysiological alterations of WML. Current findings reveal that WML is a broad concept involved multiple origins, such as genetic predisposition, age-related susceptibility, vascular anomalies, infection and toxication. Among them, the ischemia is assumed to be the most predominant cause of WMLs [2], and cerebrovenous disorder related WML, as a newly recognized WML subtype, is still under research to date. Literatures describing significant insights into the pathophysiological underpinnings of WML are also available, such as hypoxia-ischemia, endothelial dysfunction, blood-brain barrier disruption and infiltration of inflammatory mediators or cytokines [3, 4]. However, none of them can entirely explain the whole dynamic process of WML formation, posing a challenge to conduct more intensive research in this field. In addition, evidence indicates that WMLs are closely linked to clinical deficits, such as headache, mobility disorder and even cognitive impairment [5, 6]. Notably, the association of vascular WMLs and intellectual impairment is currently a concerning territory in neurodegenerative disorders and aging.

Given the facts above, this review aims to improve the understanding of pathogeneses and imaging features of WMLs in cerebrovascular diseases. Despite the arterial WMLs are reported widely, this review mainly focuses on the cerebrovenous anomaly related WMLs. For this purpose, we firstly present current knowledge across the anatomic features, neuroimaging characteristics and clinical relevance of vascular WMLs. Then, we discuss the potential pathomechanisms that involved in vascular WML formation and progression. Moreover, the potential association between vascular WMLs and cognitive decline is also briefly reviewed in this study. We outline some future investigational directions as well and hope this review will spark more studies in the future.

## 1.Normal white matter

### 1.1 Anatomic structure

**Figure 1.**
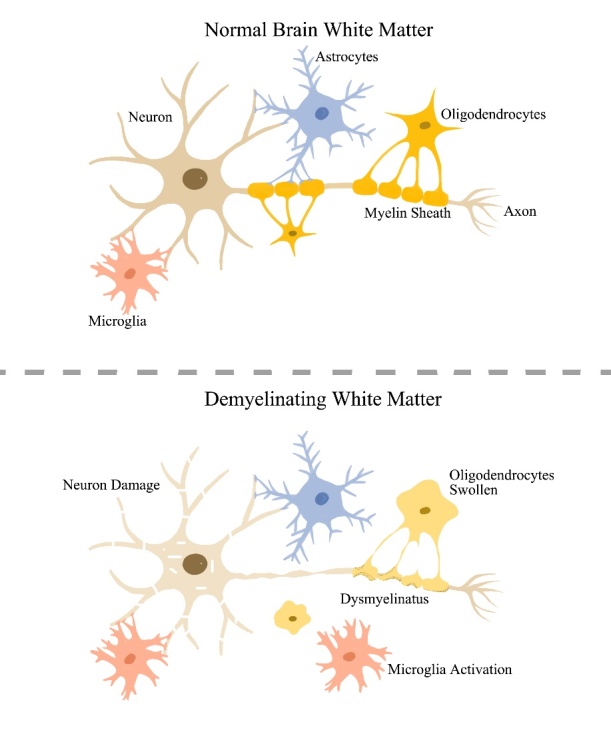
A sketch drawing of central nervous system. Top: The myelinated neurons coupled with various glial cells composed of the NAWM. Bottom: The neuron damage, demyelination, oligodendrocyte edema, and microglial activation all may contribute to the WML formation. Note: NAWM indicates normal-appearing white matter; WML indicates white matter lesion.

Both neurons and glial cells are components of the central nervous system, in which, oligodendrocytes, a subtype of myelin-producing glial cells, form the myeline sheath to surround and protect axons [7, 8]. Anatomically, as displayed in Figure 1, the myelinated axons and glial cells (astrocytes, oligodendrocytes, pericytes and microglia) are morphological and functional coupled, constituting jointly as the cerebral white matter (WM) and holding accountable for the normal appearance of WM. Any kind of damage in WM composition (for example, myelin pallor, demyelination, axonal loss, gliosis, oligodendrocyte apoptosis and edema) may change the normal structures of WM and result in WM hyperintensities on magnetic resonance scans [9].

### 1.2 Blood supply

WMLs are typically divided into periventricular white matter (PVWM) and deep subcortical white matter (DWM) lesions [10], as the vascular anatomy and pathogenic mechanisms are different in these two areas. In detail, the blood supply in PVWM area primarily comes from the ventriculopetal medullary and/or penetrating branches, which are terminal arteries almost devoid of collaterals. In this regard, PVWM is believed to be the most vulnerable area to circulatory under-perfusion [10-12]. Prior evidence showing that carotid atherosclerosis, a major contributor to cerebral hypoperfusion, preferentially predisposed to PVWMLs but not to DWMLs was consistent with such a blood supply feature [13]. Conversely, the blood supply in DWM area includes many short branches arising from the long penetrating arteries and harbors anastomoses among the feeders [11, 14], which on the one hand confers some protection to the DWM area against hemodynamical insults; on the other hand, renders the DWM more vulnerable to small vessel diseases, since these small-sized branches and anastomoses are the core substrates for fibrohyalinotic changes with wall thickening and luminal narrowing [10].

The differing vascular supply and lesion pre-disposition between PVWM and DWM further suggest that the WMLs are vessel-related events. Particularly, it is noteworthy that U-fibers, the strips of juxtacortical WM connecting the adjacent cortex, are nourished by dual blood supply: the long penetrating medullary branches and the shorter cortical arterioles, and are thus mostly escape from the hypoperfusion-related injuries [11], which further portends the potential correlation between inadequate perfusion and WML occurrence.

## 2. White matter lesion

### 2.1 The imaging modality

The good performance of FLAIR in WML detection was introduced by former studies [15-17]. In details, the FLAIR sequence can suppress the cerebrospinal fluid (CSF) as dark signals, while preserve the WM edema or lesions as brighter signals than those of normal WM, whereby providing insight into WM structural changes. However, the FLAIR technique may overestimate the WML extent due to the partial volume effects and/or the fuzzy WML boundaries [16], and be prone to false positives for the hypertense artifacts (i.e., WM edema) [15]. In contrast, the T2WI enables the simultaneous enhancement of both CSF and WMLs, co-presenting as hyperintensities [15]. Hence, using the T2WI alone is unable to differentiate WMLs from some misleading signals, such as the enlarged perivascular (Virchow-Robin) spaces, which contain CSF and appear as hyperintensities on T2WI as well [15, 18]. As described above, although both FLAIR and T2WI can exhibit the hyperintense WMLs, the extent of WM hyperintensities may not be the same on such two sequences. And the complementary usage of FLAIR and T2WI will optimize the WML detection and reduce false positives [19].

However, accumulating evidence claimed that subtle WM tract disintegrates might have developed in a step-wise manner preceding the visible WMLs on MRI maps, and novel imaging sequence, such as diffusion tensor imaging (DTI), could shed light for such tiny WML research [20]. Briefly, DTI studies can map the microstructural WM that is unable to be imaged by conventional MRI technique, through measuring the directionality and rate of the water mobility that typically enabled by the fractional anisotropy (FA) and mean diffusivity (MD) [21]. The status of WM integrity can be inferred from DTI-assisted measures, since the water molecules tend to move more rapidly along the axonal orientation and the aligned tracts on DTI-based scanning mostly represent the primary direction of axons in the brain [22, 23]. Therefore, DTI maps are able to provide details regarding the integrity of WM tracts at the microscopic level, as the diffusion signals may be imprinted once the displacement of water molecules is interrupted [24]. Of note, the earlier WM changes reflected on DTI maps may be reversible, which occur prior to but may progress into demyelination and axonal damage if not treated timely [21]. Besides, the altered microstructures identified in the normal-appearing WM (NAWM) areas may be the causes of memory decline, cognitive impairment, late-life depression and so on [25, 26]. It is hence of particular significance to recognize the invisible WM changes earlier aided by the DTI techniques, to thwart or reverse further WM damage in the sense of its clinical impacts.

### 2.2 Fazekas scale for WML evaluation

**Figure 2.**
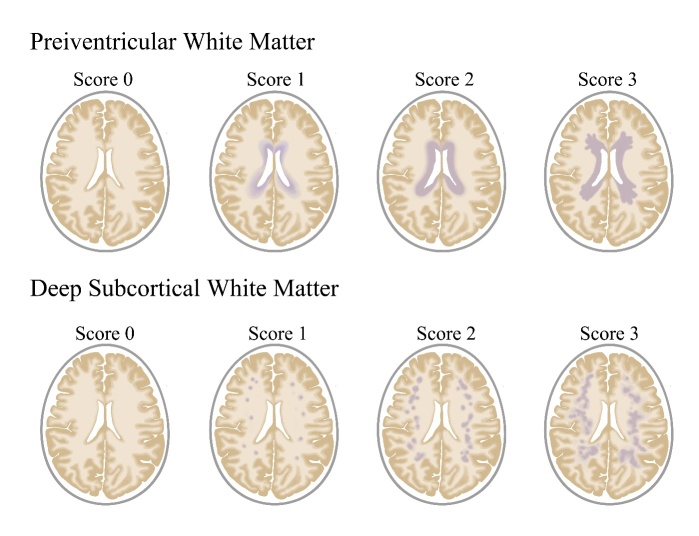
The schematic diagram of Fazekas scale to semi-quantitatively quantify the WML severity. For PVWM: no lesions, score 0; caps or pencil-thin linings, score 1; smooth halos, score 2; irregular lesions extending into the DWM, score 3. For DWM: no lesions, score 0; punctate foci, score 1; early confluences, score 2; confluences, score 3. Note: PVWM indicates periventricular white matter; DWM indicates deep subcortical white matter.

WML is a group of heterogeneous diseases with diverse pathologies, clinical course and therapy options that are considered highly related to the severity and location of lesions, and more specifically, to the lesion patterns [27]. Several visual rating scales have been devised to assess the severity of WMLs based on MRI maps [28]; among which, the Fazekas scale, first brought forward in 1987, is the most widely used one in clinical settings due to its convenience. A graphic illustration of Fazekas scale is shown in Figure 2. According to Fazekas scale, the PVWM and DWM are scored separately on two 3-point criteria. PVWML assessment: no lesions (score 0), caps or pencil-thin linings (score 1), smooth halos (score 2) and irregular signals extending into the DWM (score 3); DWML assessment: no lesions (score 0), punctate foci (score 1), early confluences (score 2) and large confluent lesions (score 3). The overall degrees of WML are equal to the sum of PVWML and DWML scores (score range 0-6) [29].

In fact, the heterogeneity in WML patterns may partly indicate different etiologies and histopathologic correlates. Concretely, the periventricular caps or pencil-thin linings and smooth halos may evolve from a similar histopathological pattern that associates with demyelination, myelin pallor, discontinuity of the ependymal lining and subependymal gliosis [10, 30]. Intracranial hypertension and other systemic diseases may possibly induce hypoperfusion and hypoxia of the entire brain, instead of local cerebral ischemia, and are usually responsible for their formation [10, 30]. In contrast, irregular periventricular hyperintensities and DWM abnormalities are prone to associate with patchy myelin rarefaction and tissue necrosis around the perivascular spaces, in which the local vascular ischemia appears to play a dominant role. Specifically, irregular peri-ventricular lesions are more likely to result from chronic hemodynamic ischemia, whereas microangiopathy is more pronounced in DWMLs [10]. Besides, punctate lesions are more likely characterized with mildly ischemic tissue damage caused by thickened arteriolar walls, while early confluent and confluent lesions in DWM are frequently determined by more extensive and complete tissue damage attested to the ischemic insults [30].

### 2.3 Risk factors and clinical significance of WML

A plethora of observations concluded that WMLs were increasingly prevalent in the elderly with a detectable ratio at about 90% among individuals over 60 years in general population [31, 32]. Besides, WML may also progress if exposure to hypertension, diabetes mellitus (DM) and other vascular risky profiles [33]. Among which, hypertension mainly imposes hypoxia-ischemia and hypoperfusion on cerebral WM, as a corollary of microvasculature structural lesions caused by the mechanical stress of hypertension, which is also the reason why hypertensive individuals are likely exposed to greater WML volume [34]. Yet only a relatively weak association is found between DM and WMLs [35, 36], although diabetes can also predispose to cerebral vascular disorders. And the excessive inflammation, activated oxidative stress, as well as the consequent dysfunction of endothelia and vascular smooth muscle cells are the proposed alterations in diabetic vessels [36, 37]. Importantly, it is possible that the well-control of these modifiable factors will to some degree prevent the WML towards progression. In support of this, a large longitudinal study has been conducted and concluded that the chance and severity of hypertension-related WMLs could attenuate with the blood pressure being well controlled [38].

In clinical situation, WMLs are closely related to various geriatric disorders, such as cognitive decline, dementia and stroke [31], albeit their great clinical relevance, current evidence as to their precise pathological mechanisms are still inconclusive, possibly due to its complex and multifactorial pathologies. In this review, the presumed WML pathogeneses and patterns arising from vascular origins, including large-artery stenosis, small vessel disease, cardiogenic embolism, cerebrovenous disease and arteriovenous fistula, will be particularly outlined with the exclusion of WMLs secondary to neuroinfection, neurodegeneration, neoplastic or toxic origins and so forth.

### 2.4 Vascular pathology and WML in the aged brain

Cerebrovascular pathological changes and WMLs may positively correlate with the dynamics of aging. Studies have detailed the large artery stiffness in the aged brain, which featured with the loss of elastin and medial muscle fibers that gradually replaced by the stiffed collagen [39, 40]. Under physiological condition, the rich contents of elastic tissues allow large arteries to buffer the pulsatile flow generated by the heartbeat. Apparently, when large arteries become stiffened with elastin fatigue and accumulated collagen, the subsequent pulsatile flow in downstream vessels may induce microvascular alterations and escalate hemodynamic stress instead. Besides, excessive flow pulsation is regarded as the condition involving the endothelia-based oxidative stress and a stimulus to endothelial dysfunction [41, 42]. Aging is also known to change the structures of cerebral arterioles, probably through the elongation of vessels and the compromised elastic fiber integrity, thus leading to arteriolar tortuosity [43]. Interestingly, investigators found that the WM tissues around the tortuous arteries were frequently lost and the arterial tortuosity was more apparent in WML areas [44]; such findings further lent weight to the possible relationship between arterial tortuosity and lesions. The microstructural changes of capillary beds in the grey population may play a pivotal role in age-specific WMLs. Degenerative capillaries may develop with disrupted microvascular integrity, decreased number of endothelial cells, perivascular collagen depositions and the resultant basement membrane thickening during the aging process [45]. Cerebral endothelial dysfunction is considered paralleled with such an altered microvasculature, which may be accountable for the impaired endothelial-dependent vasodilation [46]. In addition, endothelia dysfunction is also the cause of BBB damage that renders the blood constituents leak into the WM areas and damages the WM structure with visible lesions [47]. Aging-related vascular modeling occurs in cerebral venous system as well, characterized by the collagenous thickening of venous walls with narrowed and even occluded lumen, so as to maintain the venous tensile strength to adapt the increased arterial pulsatile pressure caused by age-associated arterial stiffening [46]. Clearly, the incidence of internal jugular venous reflux (IJVR) also increases with natural aging [48], partly owing to the age-related degenerative changes of venous valves. Both venous collagenosis and IJVR may induce WML formation and accelerate its burden accumulation in the elderly [44, 48].

Building on the present evidence, it was suggested that the prevailing neuroimaging features of age-related WMLs encompassed PVWM caps or lining lesions and punctate DWM changes, which could be observed in more than half of asymptomatic elderly [27]. Besides, frontal and parietal lobes were in lesion predominant as ever observed in normal elderly [32]. Surprisingly, symptomatic variability may associate with the lesion locations. For instance, lesions around the frontal horns are likely the detriments of executive disability in the healthy elderly, and those around the posterior horns may be the culprits of memory decline [49], whereas subcortical lesions are more pronounced in late-onset depression [50].

## 3. Cerebroarterial disease and WML

### 3.1 Large-artery stenosis and WML

Large-artery stenosis (LAS) is defined as more than 50% stenosis or occlusion of intra- or extra-cranial arteries [51]. One case-control study deciphered the scattered and round, patchy or fused lesions with clear-defined margins arising as a result of arteriostenosis [52]. Yet, there existed incongruency results among the studies regarding the interplay between WMLs and LAS.

### 3.1.1 Intracranial large-artery stenosis and WML

Intracranial large-artery stenosis (iLAS) is a group of diseases associated with cerebrovascular ischemic events enabled by varying mechanisms [53]. Abundant literatures showed that hemodynamic ischemia secondary to iLAS, irrespective of single or multiple stenoses, may contribute to WML formation in both stroke-free and stroke patients [54-56]. Indeed, vascular hazard factors, such as greater age, hypertension and probably the DM, which can themselves result in damaged WM independent of arterial stenosis as delineated above, often coexist with LAS. With iLAS per se and accelerated by the preexisting hazard factors, it is therefore not surprising that iLAS has long been linked to the higher WML risk. Paradoxically, an earlier study based on a Chinese population with stroke failed to find the association between iLAS and WML formation [57]. Perhaps a cross-sectional design and a relatively small sample size may account for such conflicting outcomes.

### 3.1.2 Extracranial large-artery stenosis and WML

The relationship between extracranial LAS (eLAS), especially the carotid artery, and WML severity remains controversial. Chutinet et al. implied that extracranial carotid artery (ECA) stenosis might impair cerebral perfusion and was to blame for the WML deterioration after eliminating the influence of iLAS [58]. Likewise, a similar finding was reported on one earlier observational study consisting that ECA stenosis might compromise the cerebrovascular autoregulation and add harm to the WMLs [59]. However, some studies failed to replicate the positive association between ECA stenosis and WMLs. For example, a large cohort study performed by Potter et al. indicated that ECA stenosis had little effect on WML load, if any, a third associated mediating factor, such as advanced age, hypertension or DM, might be accused [60]. One possible explanation for these inconsistencies may be that most of them came from cross-sectional and retrospective studies.

In corroboration of the above, existing evidence regarding the deleterious effects of LAS on WML burden remains obscure and confirmatory investigations are warranted. First of all, LAS itself may limit the blood flow in WM areas that nourished by the distal small arteries, since large arteries are interconnected with small arteries or arterioles and the blood flow is transported from the former to the latter, which can be supported by the recovered cerebral perfusion in WM regions followed with the carotid endarterectomy [61]. Actually, this insight was first illustrated by Fisher et al. in 1979 in one case series that penetrating branches extending from the middle cerebral artery (MCA) were occluded by the unstable atheromatous plaques of MCA through the artery-to-artery embolic mechanism [62]. And our previous study lent support to such a theory showing that cavities could be generally found in patients with cerebral LAS [52]. These findings indicate that upstream vessels can act on those downstream, namely the concept of ‘‘large and small artery cross-talk.’’ Conversely, a dramatically different explanation proposed by Masawa et al. for their negative association was that the large artery atherosclerosis, be it intra- or extra-cranial, could protect cerebral small arteries from medial smooth muscle cell necrosis for the above-normal serum cholesterol and the lesser tensile stress in vascular walls, as well as the reduced intraluminal pressure that followed [63]. Besides, the reduced blood flow due to LAS may closely relate to the exhausted cerebrovascular reactivity (CVR), an ability in response to vasodilatory stimulus in ischemic zones for blood flow redistribution and ischemia resistance [59]. The impaired vasodilatory capability is identified significantly attributable for the heavier WML burden, as it may lead to the reduction of cerebral perfusion [59, 64, 65]. In general, the lesions located at the periventricular walls are preferentially precipitated by impaired CVR, since the WM in periventricular walls is irrigated by the terminal branches with scarce or absent anastomoses and thereby more sensitive to hemodynamic instability [66].

### 3.2 Cerebral small vessel disease and WML

The term cerebral small vessel disease (CSVD), as suggested by its name, was primarily used to denote the pathological changes of small arteries, arterioles, capillaries, small veins and venules, but now, it is more often referred only to the pathological alterations of arterial origins, for which, another term “arterial small vessel disease” was once raised by Pantoni et al. to rename this disorder [67]. While the venous component is described as venous collagenosis [68], which will be introduced in more detail after, and now we return to the arterial part first.

CSVD refers to a broad category of complex diseases with intricate mechanisms and is classified into six types accordingly [67]. Of these types, arteriolosclerosis (type 1) and cerebral amyloid angiopathy (CAA, type 2) are known to comprise the most CSVD cases. Clinically, CSVD is known as a second leading cause of cognitive decline and dementia after Alzheimer’s disease in the elderly and a key reason for stroke-prone [67, 69]. Radiologically, compared to large-artery disease, small vessels are too tiny to capture their underpinning pathological alterations on conventional MR angiography, and CSVD diagnosis is mainly based on the interpretation of parenchymal lesions presented on neuroimaging [70]. Notwithstanding the heterogeneity in etiologies, CSVD of varying types share homogeneities in neuroradiologic markers, covering the WMLs, lacunes, cerebral microbleeds (CMBs), enlarged perivascular spaces (EPVS) and brain atrophy. Considering these markers themselves are individually associated with certain clinical impacts, recently, a total CSVD score was proposed to capture the global CSVD burden based on the combined occurrence of 4 MRI-detected markers as followed [71]: (1) irregular PVWML extending into the DWM (Fazekas score 3) or confluent DWML (Fazekas score 2 or 3); (2) lacunes; (3) CMBs; and (4) moderate to severe (>10) EPVS in the basal ganglia. One point is allocated to each of these indicators in this score, creating an ordinal scale ranged from 0 to 4 points. Previous analyses have tried to prove that the CSVD score might predict the initial and recurrent stroke risk in the ischemic population [72, 73], and cognitive decline in the diseased population [74-76]. In line with these literatures, a community-based study with long-term follow-up established that a CSVD score of 3-4 may be predictive of a higher risk of stroke events and dementia [77]. However, a recent research held the view that this score may not have additional predictive value in stroke outcomes as compared with the usual predictors (i.e., age and baseline NIHSS) [78]. Integrating these findings, the CSVD score may have practical use in assessing the clinical prognosis, yet its cutoff points that relate to the prevalence of stroke or dementia need deeper exploration and should be a priority for future research.

It is worthwhile to mention that WML is the most prominent one among the radiological evidences of CSVD [4], frequently ranging in severity from spots, patchy, to almost-confluent or confluent hyperintensities and arranging symmetrically in bilateral hemispheres [3]. Nevertheless, the pathogeneses underlying the CSVD-induced WMLs are as-yet unclear. We will take the two major CSVD types, the arteriolosclerosis (type 1) and the cerebral amyloid angiopathy (type 2), as representative examples to review the CSVD-associated WMLs in the following paragraph.

### 3.2.1 Arteriolosclerosis and WML

Type 1 CSVD, caused by arteriolosclerosis, whose prevalence is strongly associated with advanced age and hypertension, is featured with the fibroid necrosis and lipohyalinosis in vascular walls [3]. First and foremost, such arteriolar changes likely predispose the lumen of small vessels to progressive stenosis or even occlusion with reduced cerebral blood flow (CBF) and extensive tissue ischemia, whereby leading to varying degrees of myelin loss, astrogliosis, oligodendrocyte and axonal necrosis [79]. However, contradictory viewpoint went that the WMLs may be the cause rather than the result of the reduced CBF, since the lesion possibly formed prior to the reduced CBF in the affected tissue [80]. An illustration for such a finding was that CBF was less needed to supply nutrients and remove wastes in the damaged WM areas, where normal tissue was reduced and hence in low metabolic demand [80, 81]. Secondly, the arteriosclerotic conditions may cause the small vessels lose elasticity and become stiffened and unreactive, hence being unable to dilate or constrict in response to the hemodynamic variations, that is, the reduced cerebrovascular reactivity (CVR) [82]. Importantly, the existence of CVR deficits might precede the formation of lesions, and areas with impeded CVR had higher frequency to occur WMLs as observed by Sam and associates [65, 83]. These findings lent support to the notion that impaired CVR may act as an etiologic role in the WM diseases, and can be interpreted that reduced CVR may expose the WM to hypoperfusion due to its inability to preserve blood supply [65, 83]. Last but not least, it appears that the blood-brain barrier (BBB) high-permeability is also an indispensable event in the cascade of small vessel pathology [84]. Clearly, the vascular lesions of CSVD may bring harm to the BBB architectures, including the endothelial cells, cellular tight junctions (TJs), adhesion molecules and basement membranes, hence lead to the breaches of BBB with inevitable leakage of blood components into the WM and cause diffuse tissue damage [85, 86]. There was evidence suggesting that the NAWM around the visible lesions may also suffer from the elevated BBB permeability and be seen as candidates for future lesion growth [87]. Notably, as arteriolosclerosis has a strongly hypertension-related prevalence, type 1 CSVD is also named hypertensive vasculopathy [67]. Moreover, anti-hypertensive medications have proved effective in protecting the baseline WML against expansion [88].

### 3.2.2 Cerebral amyloid angiopathy and WML

Type 2 CSVD, namely cerebral amyloid angiopathy (CAA), is an age-associated vasculopathy characterized by deposits of vascular β-amyloid (Aβ) in cortex and meninges [89]. It may possibly occur owing to the loss of smooth muscle cells and the impaired Aβ clearance in the vascular media and adventitia with increasing age [90]. Ample evidence has established a causal link between CAA and WMLs [91, 92], presumably being the result of the interaction of several mechanisms. Additionally, early studies have emphasized that Aβ peptides are disproportionately prominent in occipital lobes, where the CAA-related WMLs also tend to be the most severe, hinting that vascular Aβ may exert an effect on the WM [89, 93]. This idea was supported by a finding that CAA-related WML level is dependent on the Aβ burden, raising the surmise that Aβ burden may be a predictive surrogate for CAA severity [92]. The mechanisms that mediated Aβ-associated WMLs may include the following: Firstly, vascular amyloid deposition admixture with the smooth muscle loss may impair the vascular structural integrity and result in Aβ-related vascular dysregulation [94, 95]. Evidences concerning the Aβ-provoked vascular dysfunction are widely available, including both animal and human studies. To be detail, animal literatures have deduced that Aβ proteins may lessen the adhesion between the vascular smooth muscles and basement membrane [95], and capillary Aβ may lead to the vascular occlusion followed with the reduced CBF [96]. Besides, Aβ peptides may increase cerebral susceptibility to ischemic damage because of the defective CVR, as found in a mice study [97]. These observations were corroborated by a human study showing that the Aβ-mediated vascular toxicity may decrease the vascular autoregulation and contribute to the reduced CBF and cerebral ischemia [98]. On top of the above, also worth mentioning is that Aβ may increase the BBB permeability through the detrimental effects on the tight junction (TJ) proteins as observed in both rat models and humans [99, 100]. Normally, the BBB permeability is maintained by the endothelial cells that are bound together by the TJs, whose injury may therefore lead to the imperfect BBB structure.

To sum up, the CSVD-associated WMLs are mainly caused by ischemia, CVR deficits and BBB disintegrity. Of particular note, these pathomechanisms combine together, rather act separately, to mediate the WML formation and development; and the endothelial dysfunction may function as a central role in the cascade of WML pathogeneses, since its association with both impaired CVR and BBB [101]. Despite the certain similar mechanisms, the WML distributions between arteriosclerosis and CAA are remarkably dissimilar: lesions located around peri-basal ganglia are highly associated with arteriolosclerosis, whereas multiple subcortical spots are strongly indicative of CAA [102]. Equally important, distinct topographic distributions of CMBs are also identified that arteriosclerosis is typically involved with deep CMBs in the basal ganglia, thalamus and brainstem, whilst CAA is relatively more associated with lobar CMBs [103]. Aside from WML and CMB, the other CSVD-MRI markers will not be further mentioned in this review for their irrelevancies to our major topic. If interested, other reviews can be referred to [104, 105].

**Figure 3.**
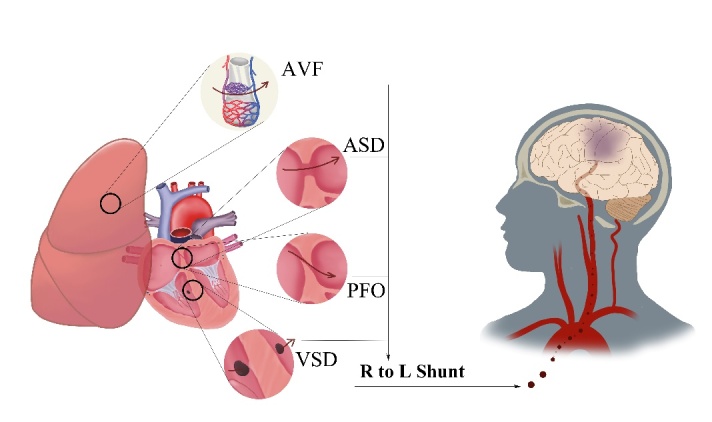
The common causes of right-to-left shunt. The PAVF, ASD, PFO, and VSD are the common entities of RLS. PAVF is a direct communication between pulmonary artery and vein without the mediation of capillaries. ASD refers to a window on the atrial level. PFO is an anatomical defect between septum primum and septum secundum. VSD is defined as a direct pathway between two ventricles. In these settings, venous micro-emboli can directly enter into to the cerebral arteries, resulting in subclinical WMLs or even cerebral infarctions. Note: PAVF indicates pulmonary arterio-venous fistula; ASD indicates atrial septal defect; PFO indicates patent foramen ovale; VSD indicates ventricular septal defect; RLS indicates right-to-left shunt; WMLs indicate white matter lesions.

**Figure 4.**
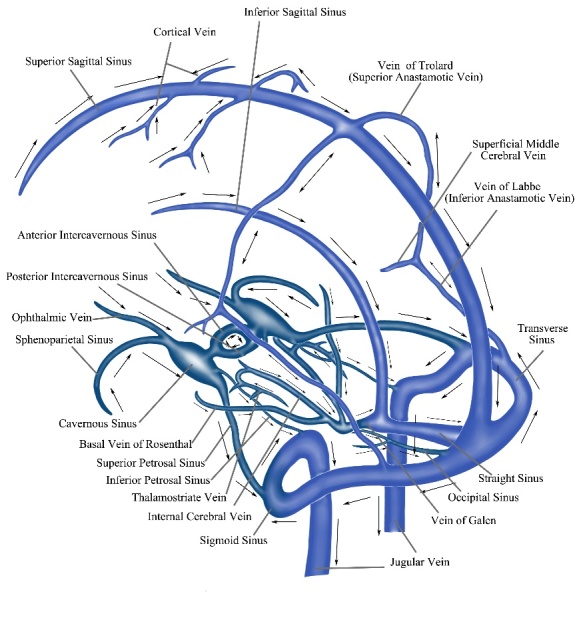
The anatomy of cerebral venous system. The superficial venous blood often outflows through the right-side transverse sinus and internal jugular vein, whereas the deep venous blood often outflows through the left side.

### 3.3 Intracardiac right-to-left shunts and WML

Intracardiac right-to-left shunt (RLS), as a potential embolic source, is commonly acknowledged as an anatomical predisposition that linked with a bulk of pathological conditions, such as cryptogenic stroke [106], migraine with aura [107] and decompression sickness [108]. Paradoxical embolism is a contributory mechanism of intracardiac RLS that allows the venous emboli to circumvent the pulmonary system, directly pass through the arteries and then into the cerebral circulation via a preexistent venous-to-arterial shunt, possibly a patent foramen ovale (PFO), atrial or ventricular septal defects (ASD or VSD), or a pulmonary arterio-venous fistula (AVF) [109] (Fig. 3). Among them, PFO is established as the most common one with an incidence of about 27.3%, as revealed by an earlier autopsy study [110]. Plenty of studies suggested that the scattered juxtacortical spots on T2WI and FLAIR maps were usually the surrogate predictors of cardiogenic embolization and could be interpreted as silent or subclinical lesions without ischemic symptoms [18, 111, 112]. Interestingly, the number of juxtacortical spots are thought to be positively correlated with the magnitude of shunting [111, 112]. A feasible explanation for this concordance is that when the degree of RLS increases, the quantification of micro-emboli that travels through the RLS will correspondingly be larger, thereby imparting an increased embolic risk [113-115]. Moreover, the wider PFO size may have an inclination to cause larger lesions for its capacity to permit the passage of larger emboli [116]. Bonati et al. also confirmed the multiple ischemic lesions to be attributable to PFO, however, he denied the association between the lesion number and the PFO size or RLS degree [117]. More interestingly, Hagen and associates implied that the PFO size seemed to have an age-related enlargement [110].

Previously, a plethora of studies have analyzed but created noises in the distribution patterns of the spotted WMLs of cardioembolic origin. For instance, some reports insisted that multiple lesions with a posterior predominance was likely a specific embolic pattern mediated by PFO [113, 115, 118]. Some studies, instead, stated that frontal lobes seemed to be the predilection site of PFO-associated embolic lesions [18, 119]; while some findings failed to identify any purported radiological signatures for paradoxical embolism [120]. The discrepancies in inspection methods and adopted instruments may be responsible for these distinct outcomes.

In regards to the pathogeneses, it is prevalently accepted that the multiple-lesion pattern may be derive of the paradoxical embolism. Indeed, venous emboli stemming from the cardiogenic RLS may impair the cerebral autoregulation, through which non-pulsate cerebral blood flow is maintained, and small thrombi are washed out under normal circumstances, and thus result in the failed emboli cleavage [121, 122]. These mechanisms interweave inextricably along with the paradoxical embolism. In precise, paradoxical embolism often leads to hypoperfusion; as a consequence, the cerebral autoregulation and emboli clearance will subject to compromise in succession owing to the insufficient blood supply.

In terms of therapy, the PFO degree to which extent that PFO closure should be performed is still debated across studies. On the one hand, two large prospective studies previously demonstrated that PFO closure may not be a necessity on the clinical ground that the presence of PFO, regardless of small or large, may not increase the likelihood of stroke if treated medically [123, 124]; rather, atrial septal aneurysm may convey the increased risk of recurrent stroke in PFO patients [125]; on the other hand, a recent meta-analysis of randomized studies showed that PFO closure was superior to medical therapy for stroke prevention, particularly in patients with moderate-to-large shunts [126]. Further well-designed and multi-center clinical trials are warranted to settle such a controversy.

## 4. Cerebrovenous hypertension and WML

Both arterial supply and venous drainage compose the cerebral circulation. Previous research mainly focused on the arterial WMLs, while the relationship between WMLs and venous diseases was relatively understudied. In anatomy, cerebral venous system includes the superficial and deep venous outflow roads, by which, venous blood in DWM and PVWM regions is respectively collected. That is, a venous watershed is present and separates the venous outflow between DWM and PVWM [127]. Venous blood from DWM and PVWM areas mostly flows out through the bilateral transverse sinuses (superficial system often toward the right side while the deep toward the left), sigmoid sinuses and then internal jugular veins (IJVs), the largest vein in the neck, and ultimately into the right atrium [128] (Fig. 4). The above-mentioned anatomy features indicate that IJV is a main pipeline for cerebral venous drainage [129]; and venous outflow hindrance caused by cerebral venous stenosis or IJVR may secondarily contribute to venous WMLs [48, 52, 130-132]. Differing from arterial WMLs, published studies have described that the symmetric, diffuse cloud-like lesions around bilateral periventricles highly suggested venous WMLs [52, 133-135]. However, the mechanisms of which still need to be elucidated.

### 4.1 Venous collagenosis and WML

The concept of “periventricular venous collagenosis (PVC)” was first proposed by Moody and associates in 1995 to describe the collagenous thickened walls of periventricular veins, which was notably correlated with WML severity [68]. From then on, the role of venous collagenosis in WML formation has been attached with more importance but yet remains unclear. On the one hand, some autopsy studies revealed that some veins, particularly superficial and intraparenchymal veins, often contained fibrohyalinous thickened and collagenized venous walls, consistent with the phlebosclerotic changes, in patients with cerebral venous thrombosis (CVT) or dural arteriovenous fistula (DAVF) or combinations thereof [136, 137]. A study on histopathology revealed that the phlebosclerotic veins in WM tissue were often accompanied by the axonal injury, demyelination plus global tissue rarefaction and even the destruction of vascular walls with blood plasma exudation [136]. These pathological changes are believed to be compatible with the manifestations of diffuse WMLs on MRI scans [136-138]. In turn, the venous collagenosis may further worsen as a corollary of protracted hypoxia-ischemia in the WM tissue caused by increased venous resistance, hence making the WM more vulnerable to lesions. On the other hand, Pettersen et al. speculated that it was the arterial diseases that resulted in tissue hypoperfusion and the ensuing excessive collagen deposition in venous walls [138], presumably based on the fact that collagenized and stenotic veins are frequently found in patients with ischemic arterial diseases [44]. And the venous collagenosis may in its turn aggravate the cerebral hypoperfusion by adding resistance to the venous system, emphasizing a bi-directional association between venous collagenosis and ischemia [138, 139]. However, this mechanism remains as a matter of speculation for its lack of more convincing evidence and calls for more thorough research.

**Figure 5.**
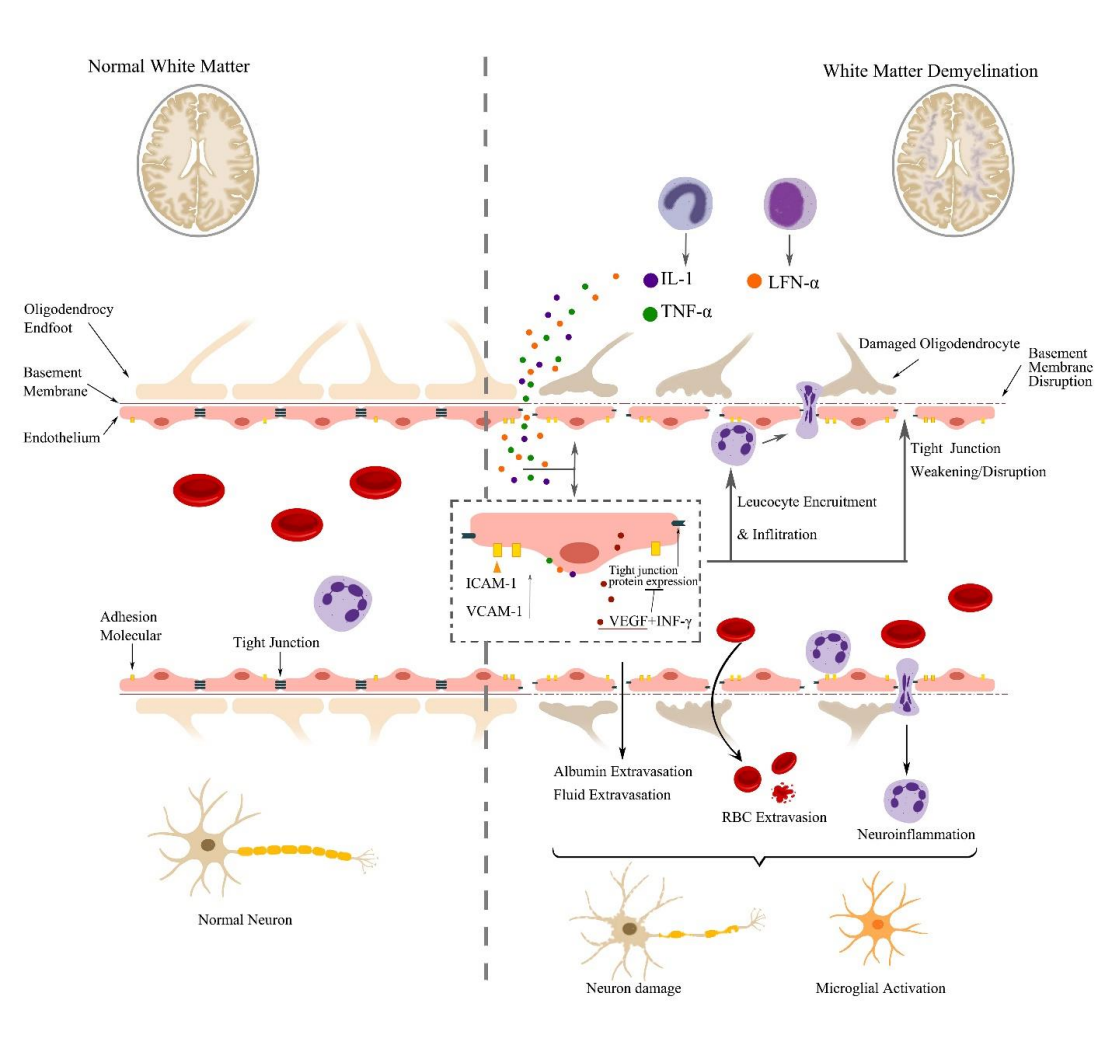
The proposed pathological mechanism of WMLs secondary to vascular endothelial inflammation.

### 4.2 Venous hypertension and WML

**Figure 6.**
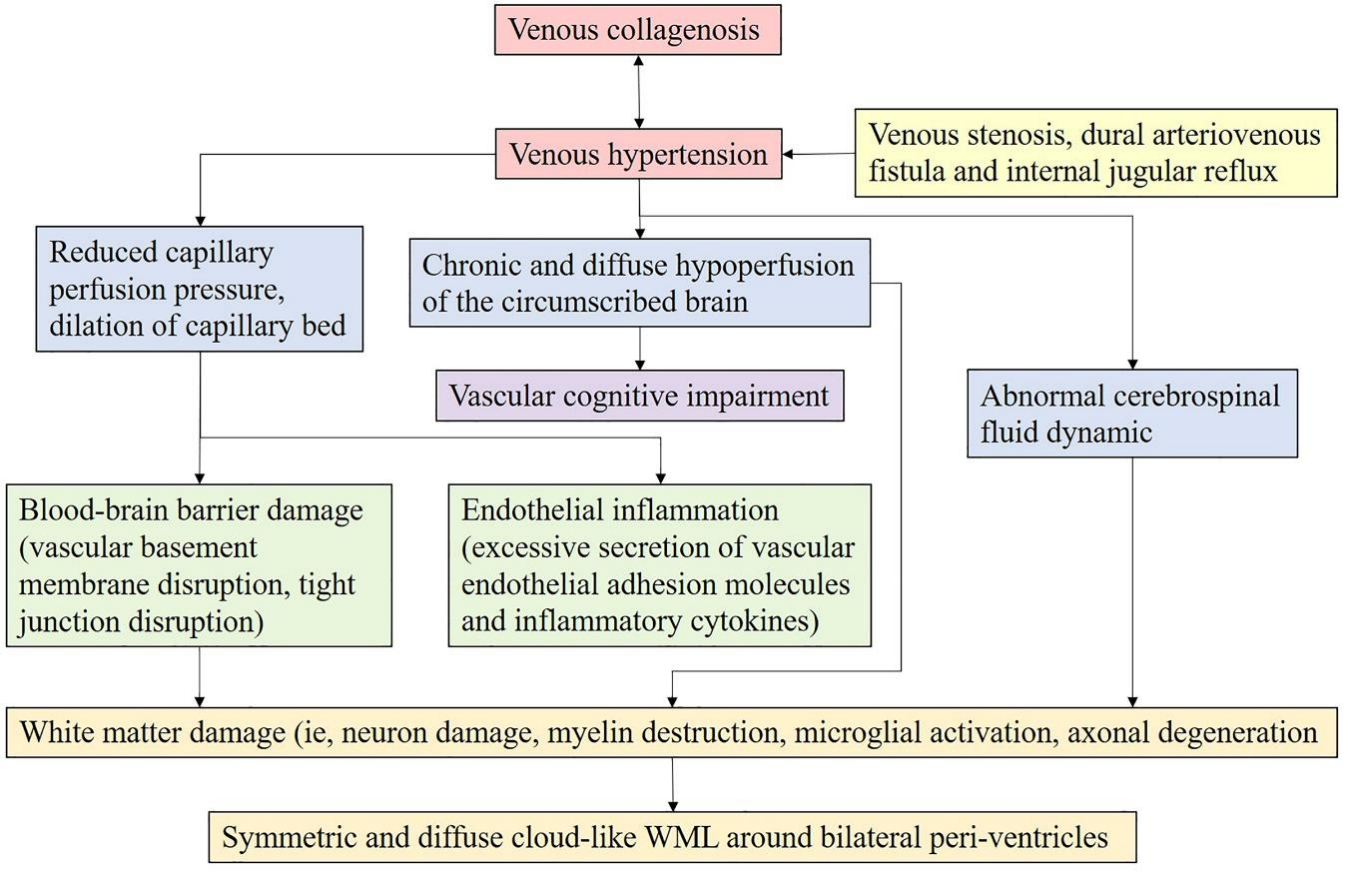
A schematic flow of proposed mechanisms underlying the venous WML formation.

According to the available literatures, venous hypertension, possibly secondary to venous obstruction or retrograde-transmitted pressure from the IJVR or DAVF, could involve in a wide spectrum of pathological changes associated with the formation of diffuse WMLs [48, 132, 134, 140, 141]. Firstly, the venous hypertension can retrograde to upstream veins, venules and capillary beds, which may induce the thickening and narrowing of the venous walls so as to compensate for the elevated pressure [142]. In this condition, the thickened venous walls may increase the vascular resistance and further worsen the venous drainage, promoting the secondary WML progression [143]. In addition, there may be an impaired venous drainage with the increased resistance of capillary perfusion in the context of venous hypertension. The venous insufficiency plus the capillary hypoperfusion may therefore work together to result in the whole-brain hypoperfusion [144]. Lastly, venous hypertension may lead to the upstream venous expansion as well, which will up-regulate the expression of vascular endothelial adhesion molecules (i.e., intercellular adhesion molecule-1, ICAM-1 and vascular cell adhesion molecule-1, VCAM-1) through activated endotheliocytes, which may be responsible for the looseness of BBB and the transmural leukocytes migration [145, 146]. It should be noted that the ICAM-1 and VCAM-1 mainly mediate the migration of leukocytes to the inflammatory sites of endothelium and only little or no are expressed by the cerebral endothelia at luminal surface under normal conditions [145], the overexpression of which may allow excessive leukocytes to adhere to the endothelia. Then the inflamed endothelia will activate the secretion of inflammatory cytokines, including tumor necrosis factor-α (TNF-α), interleukin-1(IL-1) and interferon-γ (IFN-γ), which in turn facilitate the inflammatory response and even initiate the autoimmune attacks against myelin [146]. A schematic drawing for this mechanism is present as Figure 5. Actually, venous hypertension-triggered WMLs are likely caused by the synergistic effects of more than one mechanism mentioned above, or more likely, caused by other as-yet disclosed mechanisms. A schematic flow as to mechanisms underlying venous WML formation is outlined in Figure 6.

### 4.3 DAVF-WML and IJVR-WML

Many venous disease entities, such as DAVF and IJVR, may act on symmetrical WM areas through intracranial hypertension, regardless of their different pathophysiology. DAVF, characterizing with the aberrant arteriovenous shunting between meningeal arteries and veins or sinuses [147], may contribute to diffuse WMLs through the venous hypertension created by the delivery of high-pressure arterial flow [137, 148]; surprisingly, early studies reported that the diffuse parenchymal lesions on FLAIR maps could attenuate after the fistula embolization and the venous hypertension relief [134, 137, 141]. From above, it can be hypothesized that the signal intensity of DAVF-WMLs may be paralleled with the magnitude of venous hypertension. Apart from the WMLs, a broad range of symptomology, such as the reversible vascular cognitive impairment (VCI), could also arise from DAVF [134, 137, 141, 149, 150]. It was suggested that the DAVF-induced VCI might be ascribe to the reduced CBF caused by venous hypertension, supported by the phenomenon that the recovered hypoperfusion was followed by the cognitive amelioration after DAVF embolization [134, 141]. Timely treatment might correct the radiographic and clinical anomalies in DAVF patients, and so prevent the happens of irreversible parenchymal damage and intellectual impairment that has been previously reported [141].

**Figure 7.**
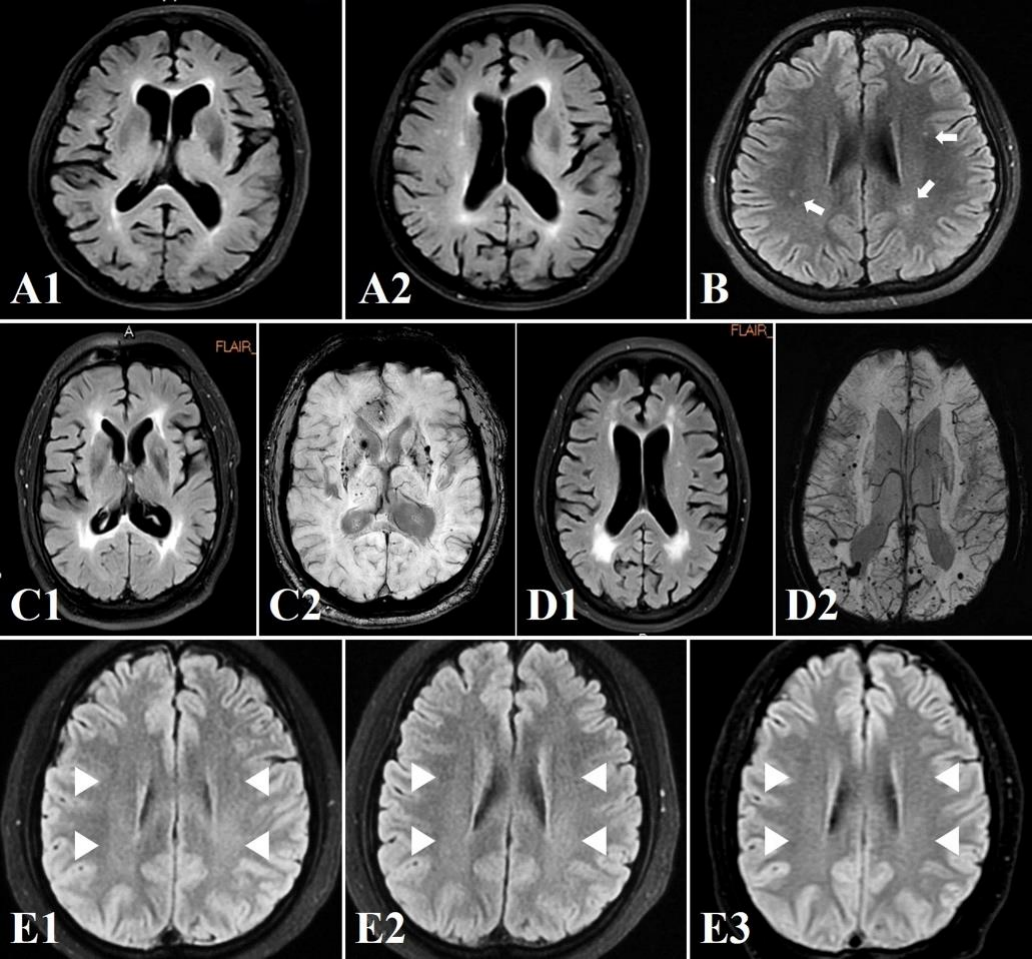
Cases of different WML patterns and the characteristic CMBs in CSVD. Age-related WMLs (A1 and A2) mainly locate at periventricular areas, especially the frontal horns; a 14-year-old girl with refractory PFO-associated migraine was found with multiple subcortical spots asymmetrically surrounding bilateral WM areas (B, white arrows); symmetrical lesions around peri-basal ganglia and periventricular horns (C1) were found in an arteriosclerosis patient with deep CMBs (C2) in the basal ganglia; multiple subcortical lesions with an occipital dominance (D1) were identified in a CAA patient with lobar CMBs (D2); venous WMLs (E1-E3) are in a symmetrical and diffuse cloud-like pattern around bilateral periventricular areas (white triangles), and reversible in selected cases as ranged in severity from E1 to E3. Note: WML indicates white matter lesion; CMBs indicate cerebral microbleeds; CSVD indicates cerebral small vessel disease; PFO indicates patent foramen ovale; CAA indicates cerebral amyloid angiopathy.

IJVR, characterized by retrograde flow in IJVs, is recognized as a cause of retrograde venous hypertension that may possibly predispose to the diffuse WMLs and VCI [48, 132, 140, 143]. High-rate IJVR in senile citizens is possibly triggered by the age-dependent structural changes in venous valves and walls [46, 151]. It is worth noting that the mechanisms responsible for IJVR-induced WMLs are similar as those for DAVF-mediated WMLs, since both IJVR and DAVF may manifest with venous hypertension, although the former possibly harbors a lesser degree as mediated by the retrograde venous pressure whilst the latter driven by the rapid arterial shunts. Recent literatures also indicated these two diseases entities shared similar features on MR sequences and may result in confused diagnosis [152, 153]. For which, we posit that they may be distinct from the WML intensity, since IJVR may harbor a milder venous hypertension for its venous origin, while the venous hypertension of DAVF may be relatively severer as created by the high-flow arterial source. Frustratedly, present studies have neither compared the WML intensity between DAVF and IJVR after the adjustment of confounding variables, nor labeled the reversibility of IJVR-associated WMLs. One reason for this condition may be the unavailable therapy for IJVR. Future studies with novel IJVR therapy are needed to confirm the validation of our hypothesis.

### Limitations and perspectives

This review has some limitations: Firstly, it only outlines the vascular origin-induced WMLs, while WMLs with other etiologies are not involved. Secondly, some mechanisms underlying the WML formation in this study have not been clearly established and warrant further confirmation. Last but not least, this review mainly discusses the qualitative assessment of the WML burden based on simple visual rating; if possible, future research can analyze the relationship between the quantitative variables (e.g., the stenotic degree, the magnitude of venous hypertension) and the precise WML volume.

### Summary

According to the studies reviewed above, PVWM is the most vulnerable area to hypoxia-ischemia, followed by DWM, while the U-fibers are relatively resistant to hypoxia-ischemia and hence often spare from the menace of WMLs. A variety of microstructural changes have been identified in aged vessels, and the periventricular caps or linings plus punctate lesions may favor the age-related WM with a frontal and parietal predominance.

The highlight in this review is that we investigate the dissimilarities in pathogeneses and neuroimaging patterns of both arterial and venous WMLs. In general, LAS-induced WMLs usually appear as well-demarcated dots or patches; arteriosclerosis-related WMLs mainly manifest as symmetrical lesions with a preference of peri-basal ganglia; CAA-associated WMLs may be not uniformly distributed, but more likely to occur in occipital lobes; multiple ischemic spots may be traces of silent cardioembolic lesions caused by paradoxical embolism; whereas, venous WMLs may feature with a symmetrical cloud-like pattern around bilateral periventricles, as showed in Figure 7.

A question regarding the precise definitions for clearly-demarcated lesions in arterial diseases and cloud-like lesions in venous diseases may hence be put forward. Frankly, their proper definitions and distinctions are lacking and retain some ambiguity; but it is generally accepted that clearly-defined lesions represent the focal ischemia created by local CBF reduction, while the cloudy lesions mean the whole-brain hypoperfusion because of the prolonged and persistent exposure to intracranial hypertension. Besides, it is of utmost importance to bear in mind that the purported radiological WML patterns mentioned in this study can only assist in predicting, rather than determining, the potential causes. Also, it should be acknowledged that our conclusions are not absolute since they are drawn based on the previous studies and the radiological patterns were not established specifically across the involved studies. Therefore, the WML pattern should be seen as a complementary diagnostic reference but not a diagnosis standard. With respect to the underlying mechanisms, cerebral arterial diseases may be mediated by hypoperfusion, embolism, endothelial dysfunction and BBB breakdown, while cerebral venous diseases are predominantly determined by venous hypertension.

Currently, the role of venous WML is emerging and its underlying mechanisms and clinical impacts may likely represent a field left for research and further answer. A majority of previous studies only focused on the role of venous hypertension functioned in cloud-like WMLs, whereas their quantitative relationship whether or not to be validity are poorly understood. Thus, an important territory warranted for in-depth investigation is the link between the degree of venous hypertension and its contribution to the intensity of WM changes.
